# Left S3 + S8 Segmentectomy with Rare Interlobar A3 Vascular Anomaly: A Case Report

**DOI:** 10.70352/scrj.cr.24-0013

**Published:** 2025-05-01

**Authors:** Hiroki Imabayashi, Takahide Toyoda, Kazuhisa Tanaka, Yuki Sata, Terunaga Inage, Hajime Tamura, Masako Chiyo, Yukiko Matsui, Hidemi Suzuki

**Affiliations:** 1 Department of General Thoracic Surgery, Chiba University Graduate School of Medicine, Chiba, Chiba, Japan

**Keywords:** left interlobar A3, pulmonary artery variants, segmentectomy, lung cancer, case report

## Abstract

**INTRODUCTION:**

Segmentectomy is being accepted as a valid operative procedure for small peripheral non-small cell lung cancer. Understanding pulmonary artery (PA) variations is essential to ensure safe and reliable surgeries. Herein, we report a case of left S3 and S8 segmentectomy involving a complete interlobar branch of the left A3, a relatively rare anomaly reported in less than 0.56% of cases in previous studies.

**CASE PRESENTATION:**

A woman in her sixties was referred to our hospital with two nodules in the left upper lobe anterior segment (S3, 1.1 × 0.8 cm) and the lower lobe anterior basal segment (S8, 1.8 × 1.7 cm), suggestive of double primary lung cancer. Preoperative thin-slice computed tomography (CT) and three-dimensional CT revealed a vascular anomaly in which the entire left A3 branched from the interlobar PA. Left S3 and S8 segmentectomies were performed via thoracotomy. The interlobar A3 branched at nearly the same level as the A6. After cutting the V3b and V3c veins, the intersegmental plane and the interlobar A3 were sequentially divided using staplers. To prevent torsion of the remaining lung, the edges of the apico-posterior segment (S1+2) and the lingular segment were loosely secured with silk sutures. The operative times were 4 h and 8 min with minimal blood loss. Pathological examination revealed that both nodules were squamous cell carcinomas of the lungs (pT1bN0M0, pStage IA2). The patient remained recurrence-free for over 1 year.

**CONCLUSIONS:**

Complete interlobar branching of the left A3 is uncommon. During left S3 segmentectomy in cases involving an interlobar A3, the S1+2 and lingular segments may become solitary blocks, necessitating measures to prevent torsion.

## Abbreviations

3D-CT
: three-dimensional computed tomography
CT
: computed tomography
NSCLC
: non-small cell lung cancer
PA
: pulmonary artery

## INTRODUCTION

Recent randomized non-inferiority trials comparing segmentectomy with lobectomy for small peripheral non-small cell lung cancer (NSCLC) have demonstrated the non-inferiority of segmentectomy.^1)^ As segmentectomy becomes increasingly established as a valid oncological option for the treatment of small peripheral NSCLC, thoracic surgeons must be well-versed in patient selection, segmentation techniques, and lymph node assessment for segmentectomy in early-stage NSCLC.^2)^ Compared with lobectomy, segmentectomy requires more precise handling of the pulmonary arteries (PAs) and veins, necessitating a thorough understanding of their anatomy.^3)^ Herein, we report a case of left S3+S8 segmentectomy for double lung cancer involving a complete interlobar branch of the left A3, a relatively rare anomaly reported in less than 0.56% of cases in previous studies.^4,5)^ To our knowledge, this is the first case report of left S3 segmentectomy with complete interlobar A3. This vascular anomaly in S3 segmentectomy raises a unique concern regarding the possibility of postoperative lung torsion. Our case report adheres to the case report (CARE) checklist.^6)^

## CASE PRESENTATION

**Fig. 1:**
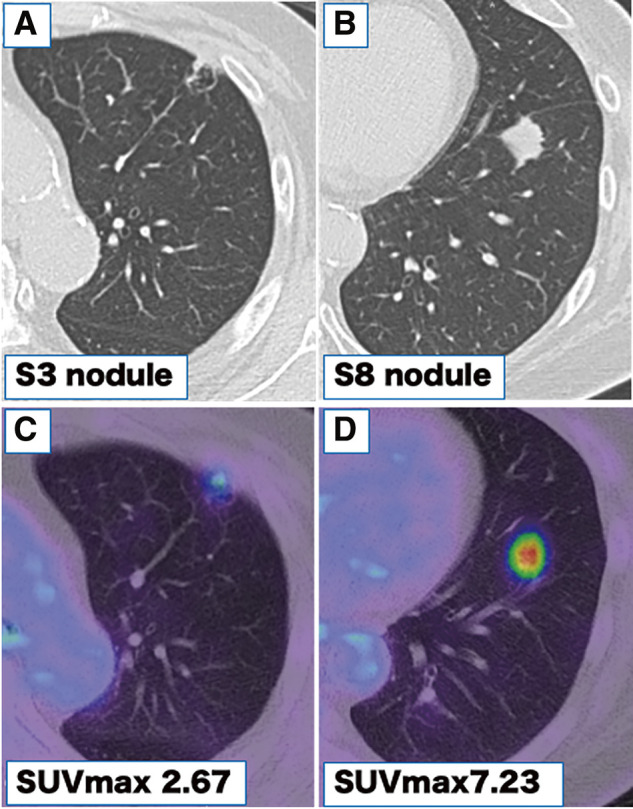
Chest computed tomography of the nodules. (**A**, **B**) Nodules measured 1.1 × 0.8 cm and 1.8 × 1.7 cm in the left S3 (**A**) and S8 segments (**B**), respectively. (**C**, **D**) 18F-fluorodeoxyglucose positron emission tomography demonstrated abnormal uptake in the tumors, with maximum standardized uptake values of 2.67 (**C**) and 7.23 (**D**), respectively.

**Fig. 2:**
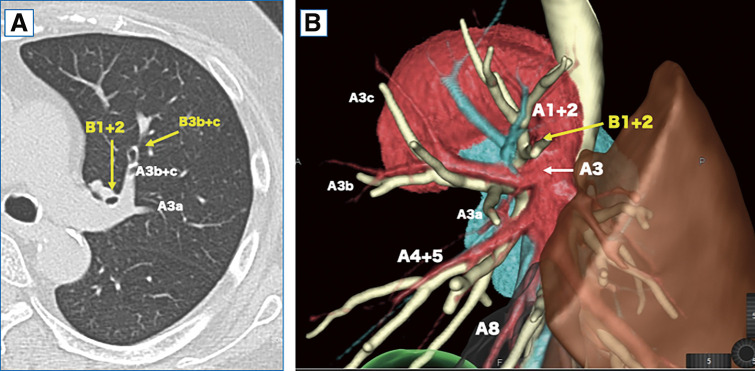
Thin-slice computed tomography (CT) and Three-dimensional CT findings. (**A**) Thin-slice computed tomography (CT) findings: The left A3 exhibited vascular branching, with all branches originating from the interlobar pulmonary artery. (**B**) Three-dimensional CT findings: The left A3a branched from the A3 stem, while A3b and A3c branched at the periphery. The lingular artery branched independently from the interlobar pulmonary artery.

**Fig. 3:**
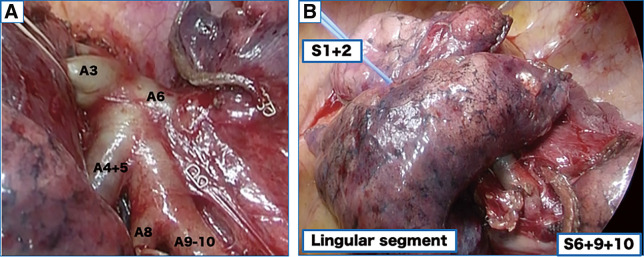
Intraoperative findings. (**A**) The interlobar A3 branched at the same level as A6 from the main pulmonary artery. (**B**) After S3 and S8 segmentectomy, the S1+2, lingular segment, and S6+9+10 were separated into isolated blocks, raising concerns about potential torsion.

A woman in her sixties was referred to our hospital with two nodules in the left upper lobe anterior segment (S3, 1.1 × 0.8 cm) and the lower lobe anterior basal segment (S8, 1.8 × 1.7 cm), detected by chest radiography and computed tomography (CT) during a medical check-up (**Fig. 1A** and **1B**). 18F-fluorodeoxyglucose positron emission tomography showed abnormal uptake in the S3 and S8 tumors, with maximum standardized uptake values of 2.67 and 7.23, respectively, leading to a surgical plan under suspicion of double primary lung cancer (**Fig. 1C** and **1D**). Thin-slice CT revealed a vascular anomaly, with the left A3 branching from the interlobar fissure (**Fig. 2A**). Preoperative three-dimensional (3D) CT was performed using Ziostation 2 and REVORAS (Ziosoft Inc., Tokyo, Japan) (**Fig. 2B**). The A3a branched proximally from A3, while the A3b and A3c branched distally. The A4/5 was of the interlobar type and branched independently of A3. No other apparent anomalies were observed in the pulmonary arteries (PAs) or the bronchi of the lower lobe. Since lung cancer was strongly suspected based on CT findings, surgical resection was performed without intraoperative pathological confirmation. A short edited intraoperative video is available in **Supplementary Video 1**. The surgery was conducted through a 10-cm axillary fifth intercostal thoracotomy, with a 2-cm incision in the seventh intercostal space for the camera port. Due to a partially incomplete fissure, the PA was exposed from the dorsal side, revealing the interlobar A3 branching the same level as A6 (**Fig. 3A**). V3b and V3c were separated from the anterior side, while A3 was separated from the interlobar side. The intersegmental plane and A3 were sequentially divided using staplers. With A3 bifurcation on the interlobar side and the V3 bifurcation anteriorly, the apico-posterior (S1+2) and the lingular segments were completely separated as blocks after S3 segmentectomy (**Fig. 3B**). An S8 segmentectomy was performed simultaneously. ND1a lymph node dissection and #10 lymph node sampling were carried out. To prevent torsion, the staple line ends of the intersegmental plane of S1+2 and the lingular segments were loosely linked using silk sutures (**Supplementary Fig. 1** and **Video 1**). The operation time was 4 h and 8 min, with 20 g blood loss. Pathological examination revealed squamous cell carcinoma in both the left S3 and S8 nodules, which were diagnosed as pT1bN0M0, pStage IA2. A prolonged air leak occurred, and pleurodesis with OK-432 was performed on postoperative day 5. The chest tube was removed on postoperative day 9. The patient remained recurrence-free for over 1 year.

## DISCUSSION

Anatomical variations are more common in the left PA than in the right PA.^7–9)^ Ma et al. reported that 97.6% of left upper lobe branches typically consist of 3–5 branches, with a minimum of two and a maximum of seven branches observed, indicating highly diverse vascular patterns.^10)^ Yamashita investigated whether the lingular artery branches from the first branch centered on the mediastinal A3 or from the second branch centered on the interlobar A1+2. They found that 72.7% (120/165) were interlobar-type lingular arteries.^11)^ Murota et al. further refined this classification of branching patterns in the interlobar region, focusing on the relationships between A1+2c, the lingular artery, and A6, into seven major categories and 85 subcategories.^4)^ In this classification, interlobar branching of A3a was observed in 8.4% (27/320) of cases, but no cases were found where the first mediastinal branch was absent and all A3 branches, including A3b and A3c, branched from the interlobar fissure.^4)^ Similar results have been reported in other studies.^4,11,12)^ Conversely, cases such as ours, where all A3a, b, and c branches from the interlobar fissure were reported, were identified by Maki et al., with 3 out of 539 cases (0.56%) presenting with interlobar-type lingular arteries.^5)^ A summary of the previously reported cases of interlobar A3 branching is presented in **Table 1**. Three-dimensional CT (3D-CT) interpretation, as demonstrated in this case, is considered a useful tool for the preoperative detection of pulmonary arteriovenous branching anomalies. Accurate preoperative understanding of the direction and changes in PAs is crucial to reduce the risk of bleeding.^13,14)^ Furthermore, in addition to evaluating PA branching patterns using 3D-CT, thin-slice CT images should be assessed to better evaluate the fine PA branches.^15)^ Even in segmentectomy cases involving rare vascular anomalies, such as the present case, careful preoperative simulation using 3D guides and thin-section CT images allowed us to perform the surgery without unexpected bleeding. After S3 segmentectomy, with the base of A3 on the interlobar side and the base of V3 anteriorly, S1+2 and the lingula were completely separated, necessitating measures to prevent torsion of the remaining lung. By predicting the relationship of the remaining lung after segmentectomy based on vascular positioning, and considering intraoperative precautions such as preserving the mediastinal and interlobar pleura as much as possible, postoperative complications can be reduced.^16)^

**Table 1:** Summary of reports on the population of the interlobar types A3a and A3

First author	Publication year	Study method	Number of study cases	ILA3a (%)	ILA3 (%)
Yamashita H.	1978	Cadaver Autopsy	165	16 (9.7)	0 (0.0)
Murota M.	2020	3D-CT	320	27 (8.4)	0 (0.0)
Maki R.	2022	3D-CT	539	54 (10.0)	3 (0.56)
Xu H.	2023	3D-CT	100	2 (2.0)	0 (0.0)
Total			1124	99 (8.8)	3 (0.27)

3D-CT, three-dimensional computed tomography; IL, interlobar

## CONCLUSIONS

We encountered a case of left S3 segmentectomy involving a highly unusual complete interlobar branch of the left A3 segment. Preoperative identification of PA anomalies through 3D-CT and detailed surgical simulations, including assessment of the remaining lung after resection, is essential for ensuring surgical safety. In cases of left S3 segmentectomy with an interlobar A3, it may be necessary to implement measures to prevent torsion, as the S1+2 and lingular segments are prone to becoming isolated blocks.

## ACKNOWLEDGMENTS

The authors thank Editage for their assistance in proofreading the English text.

## DECLARATIONS

### Funding

None.

### Authors’ contributions

TT, HI, and KT performed the surgery, and HI, TT, KT, YS, TI, HT, MC, YM, and HS followed up the patient.

The manuscript was prepared by HI and TT, under the supervision of HS.

The authors read and approved the final manuscript.

### Availability of data and materials

Data sharing is not applicable to this article, since datasets were neither generated nor analyzed for the case report.

### Ethics approval and consent to participate

Patient privacy was considered and the manuscript did not include any identifying information.

### Consent for publication

The patient provided informed consent for the publication of this case report.

### Competing interests

All authors completed the ICMJE uniform disclosure form. The authors declare that they have no conflicts of interest.

## SUPPLEMENTARY MATERIALS

Supplementary Figure 1 Intraoperative findings. To prevent torsion, the staple line ends of the intersegmental plane of S1+2 and the lingular segments were loosely linked using silk sutures (yellow allow).

Supplementary Video
