# Supplementary material for: Left S3 + S8 Segmentectomy with Rare Interlobar A3 Vascular Anomaly: A Case Report
Source: Surg Case Rep. 2025 May 1;11(1):24-0013. doi: 10.70352/scrj.cr.24-0013 (PMC12055235; doi:10.70352/scrj.cr.24-0013)

Supplemental Figure 1. Intraoperative findings

To prevent torsion, the staple line ends of the intersegmental plane of S1+2 and the lingular segments were loosely linked using silk sutures (yellow allow).

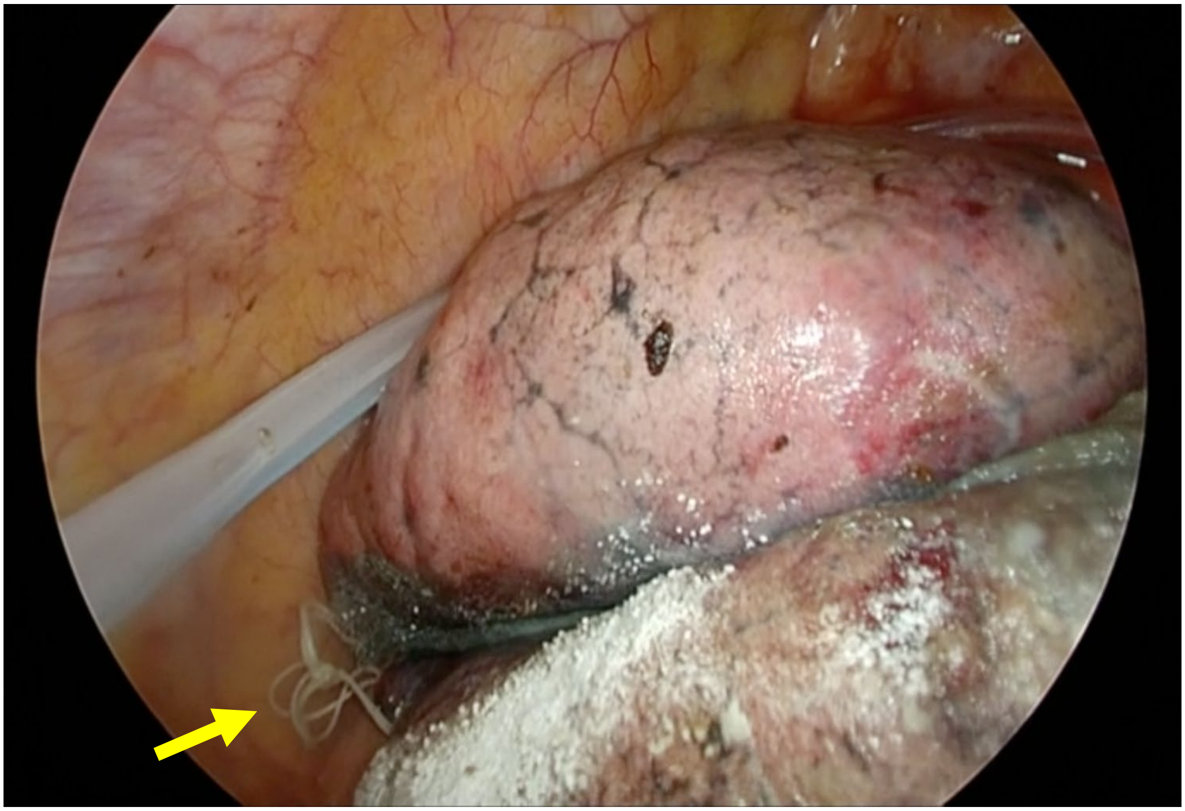

Supplement: Supplementary Figure 1 [file scr-11-01-24-0013-s001.pdf]
